# The Russian Orthodox Church as moral norm entrepreneur

**DOI:** 10.1080/09637494.2016.1194010

**Published:** 2016-07-19

**Authors:** Kristina Stoeckl

**Affiliations:** ^a^ Department of Sociology, University of Innsbruck, Innsbruck, Austria

**Keywords:** Russian Orthodox Church, norms, morality politics, religion, international institutions

## Abstract

Conflicts over religious symbols in the public sphere, gay marriage, abortion or gender equality have shown their disruptive potential across many societies in the world. They have also become the subject of political and legal debates in international institutions. These conflicts emerge out of different worldviews and normative conceptions of the good, and they are frequently framed in terms of competing interpretations of human rights. One newcomer voice in conflicts over rights and values in the international sphere is the Russian Orthodox Church (ROC), which in recent years has become an active promoter of ‘traditional values’ both inside Russia and internationally. This article studies the ideational prerequisites and dynamics of Russian Orthodox ‘norm protagonism’ in the international arena.

Conflicts over religious symbols in the public sphere, gay marriage, abortion or gender equality have shown their disruptive potential across many societies in the world. They have also become the subject of political and legal debates in international institutions.^1^ These conflicts emerge out of different worldviews and normative conceptions of the good, and they are frequently framed in terms of competing interpretations of human rights. In this article, I will look at one newcomer voice in conflicts over rights and values in the international sphere: the Russian Orthodox Church (ROC). In recent years, the ROC has become an active promoter of ‘traditional values’ both inside Russia and internationally, but the dynamics and ideational prerequisites of its ‘norm protagonism’ (see below) are still poorly understood. Scholars have either focused on the domestic situation (Agadjanian forthcoming, 2011; Stepanova 2015) or have interpreted the ROC’s international value-based agenda as an instrument of Russian soft power and foreign policy (Curanović and Leustean 2015; Anderson 2015; Laruelle 2015). With this article, I want to make a contribution to this emerging field of research by shifting the focus away from geopolitics and from the question of Russian church–state relations to an assessment of the ROC as a moral agent in the international sphere in its own right.

To do so, this article engages the literature on ‘norm entrepreneurship’, a concept used in international relations (IR) theory where it describes the normative agency of actors in transnational governance regimes. Restricting my analysis to the activity of the ROC in *international* morality politics and leaving aside deliberately the Russian domestic situation, I will present a series of cases that exemplify how the ROC acts as moral norm entrepreneur. I argue that, with its traditional values agenda, the ROC has made itself the author of a new cognitive frame for describing a specific normative position in a global controversy over the right interpretation of human rights.

## Norm entrepreneurship

‘Norm entrepreneurship’ or ‘norm protagonism’ are terms used in the study of IR to describe the normative agency of actors in transnational governance regimes. The observation behind the concept is that norms ‘do not appear out of thin air; they are actively built by agents having strong notions about appropriate or desirable behaviour in their community’ (Finnemore and Sikkink 1998, 286). The concept was developed by Finnemore and Sikkink in the context of constructivist theory in IR, which attempts to offer an alternative to rational choice models (Ruggie 1998; March and Olsen 1998). The gist of the argument is that rational choice models alone cannot exhaustively explain the intrinsic motivations of certain actors. Not only rational cost–benefit calculations motivate actors, but also norms and ideas. However, the concept deliberately plays on the rational connotations of the word ‘entrepreneur’ in order to express the idea that, once actors have made their choices, the strategies they use to pursue their goals are rational and ‘sophisticated in their means-ends calculations’ (Finnemore and Sikkink 1998, 910).^2^ A more neutral term that avoids the problem of connotations mentioned above is ‘norm protagonism’ (Sikkink 2014).^3^

Norm entrepreneurs ‘create’ norms by calling attention to issues that hitherto have not been ‘named, interpreted and dramatised’ (Finnemore and Sikkink 1998, 910) as *norms*. They construct cognitive frames, often in opposition to rival frames, effectively causing a shift in public perceptions of appropriateness. In the case of the establishment of women’s suffrage, for instance, the socially acceptable view at the time was that women were unfit for independent judgement. In the case of the creation of the International Red Cross, there was a break with the established practice of treating enemy medical personnel as spoils of war (Finnemore and Sikkink 1998). As these examples already demonstrate, most studies on norm entrepreneurship focus on progressive actors that promote norms like equality, freedom, education or welfare through international organisations such as the European Union or the United Nations, or through international nongovernmental organisations (NGOs) like the Red Cross (Keck and Sikkink 1998; Kleibrink 2011; Risse and Sikkink 1999; Sikkink 2014; Vadura 2015; on conservative norm protagonism, see Bob 2012). The ROC’s promotion of traditional values is of a different kind: it is conservative, not progressive. But despite this difference, the *mechanism* of norm entrepreneurship is perfectly comparable to the cases just cited. In order to understand how the ROC effectively creates a *new* cognitive frame called ‘traditional values’ and before looking at some examples, a closer examination of the ROC’s attitude to human rights and the Universal Declaration of Human Rights is necessary.

## Human rights against human rights

The ideological spectrum inside the ROC today is usually divided by scholars into three factions: fundamentalists, liberals and traditionalists (see Papkova 2011; Kostjuk 2005; Verkhovsky 2012).^4^ This internal pluralism and multivocality is a crucial factor for understanding the role of the ROC in Russian society today. However, for the analysis of the role of the ROC in *international* affairs, outside Russia, I would argue that one camp merits particular attention: the traditionalists.^5^ This group comprises the church’s current key leadership figures around Patriarch Kirill, many of whom come from or belong to the External Relations Department of the ROC that was formerly headed by him. The traditionalist position inside the ROC has at times been described as ‘pragmatic’ (Kostjuk 2005) rather than ideological, but this article wants to show that the traditionalists have formulated, certainly in their external relations, a precise ideological position. This position can be summarised as a ‘defence of traditional values’. Yet, in order to understand more precisely what is at stake, we need to situate the ideological stance of the traditionalists in a larger debate inside the ROC on human rights.^6^ The attitude of the ROC to human rights as an idea and a legal instrument has already been discussed at length in this journal (Agadjanian 2010; Stoeckl 2012) and elsewhere (Brüning and Van der Zweerde 2012; Namli 2014; Stoeckl 2014). The main point, which is relevant in the context of this article, is that from 2000 to 2008 the discursive strategy of the Moscow Patriarchate with regard to human rights changed. It evolved from a clear-cut rejection of human rights as a western invention to endorsing human rights as a concept, but utilising the concept in a way that was opposed to the liberal and egalitarian evolution of the international human rights system. This change was the work of the traditionalist camp inside the ROC, and it sidelined the fundamentalists, who rejected any engagement with the topic, and the liberals, who would have preferred a clearer endorsement of human rights.^7^

By ‘liberal and egalitarian evolution of the international human rights system’, which the ROC opposes, I mean that human rights in today’s world are not a static concept. Today’s international human rights system is based on the *Universal Declaration of Human Rights*, the documents and declarations of the United Nations, the *European Convention of Human Rights*, and the resolutions and documents of the Council of Europe. This international human rights system is in a constant evolution and process of interpretation. This evolution is basically one of expansion. In particular, the European Court of Human Rights (ECHR) has made sure that human rights are consistently applied to groups that have seen individual rights denied to them in the past: religious minorities, sexual minorities, but also children (Koenig 2015; McCrudden 2014). The ROC – but for that matter also conservative forces in many other countries – resent the general tendency of the international human rights regime to expand and grant freedom and equality to individuals and groups that are not, at least not in the eyes of those protagonists, representative of the traditional majority population or culture of a country. This resentment becomes clear in the following quote from an interview by the representative of the Moscow Patriarchate in Strasbourg Igumen Filip (Ryabykh) after the publication of a critical report by the United States Commission on International Religious Freedom: ‘The Russian Church believes that state and society should secure rights of all citizens and not only some of them. Minorities’ rights shouldn’t be secured at the expense of majority’s rights’ (Ryabykh 2010a). Therefore, the ROC opposes not human rights *tout court*, but the gradual egalitarian evolution of the human rights system.

It is important to remember that, when the *Universal Declaration of Human Rights* was ratified by the members of the United Nations in 1948 (when the Soviet Union abstained), gross human right violations, or what would today count as gross violations, were commonly and legally practised in most countries, even in the West: discrimination based on race, colour, religion, sex or national origin was outlawed in the United States only in 1964; marriage divorce became legal in Italy in 1970; homosexual conduct was a penal offence in Austria until 1971; and physical violence as a legitimate aspect of parental care was outlawed in Germany only in 1980. A whole range of provisions in terms of gender and generational equality, which we consider standard rights instruments today, are in fact the result of the evolution of the human rights idea and of its diffusion into national legislations.

The international human rights system by and large does not have legal and other means to ensure a country’s compliance with the obligations contained in human rights treaties after it has signed such treaties. Domestic legislative change in the current human rights system is never automatic; it is the result of domestic adaptation to a norm that has been formulated first in the international context (Risse and Sikkink 1999). The literature on norm entrepreneurship refers to this process as ‘norm diffusion’, ‘norm cascade’ and ‘norm internalisation’ (Finnemore and Sikkink 1998).

The ROC opposes this type of rights diffusion, and it does so with an argument that recalls favourably the discrepancies between human rights and legal practice that were admissible in 1948. I have previously called this argument ‘the discovery of article 29’ (Stoeckl 2014, 60–65). We find it expressed in numerous ways and by various members of the ROC. The following statement by Patriarch Kirill – the then Metropolitan of Smolensk and Kaliningrad – is representative:

I am convinced that the concern for spiritual needs, based moreover on traditional morality, ought to return to the public realm. The upholding of moral standards must become a social cause. It is the mechanism of human rights that can actively enable this return. I am speaking of a return, for the norm of according human rights with traditional morality can be found in the Universal Declaration of Human Rights of 1948. (Metropolitan Kirill 2006)

The norm referred to here by Kirill is article 29 of the Universal Declaration of Human Rights.^8^ The ‘discovery’ of this article had an important effect on human rights debates within the ROC in that it led to a new argumentative strategy.^9^ Article 29, in particular provision 29 (2), allowed the church to position itself no longer simply in opposition to a western progressive understanding of human rights, but instead to actively present itself as the vanguard of a more *original* understanding of human rights according to article 29, an understanding which emphasises the importance of morality, duties and community. The fact that the ROC during the 2000s shifted its opposition towards human rights from a principled rejection to a historical reasoned rejection may seem like a minor shift. After all, its opposition and rejection remained the same. But in terms of the church’s public agenda, this shift made a cardinal difference. It was the precondition for the ROC to become a norm entrepreneur in the field of international morality politics.

The ROC’s norm promotion is of a different kind from the norm diffusion that is usually studied by IR scholars who ask how international human rights norms diffuse into domestic legislation. Not only is it conservative, not progressive, but also the declared source of the promoted norms is different: it is, in the words of the Patriarch of Moscow Kirill, ‘traditional values rooted in all world religions’.^10^ The great novelty in this type of norm promotion by the ROC is that the *legitimacy* of this source in public debate is now said to derive from the Universal Declaration of Human Rights, specifically from article 29,^11^ and not from religion as such. The ROC couples its argument for traditional values rooted in religion with article 29 of the Universal Declaration, in order to, first, universalise the message of traditional values (since non-Orthodox people can relate to traditional values) and, second, to have it accepted into the discursive game of rights advocacy.

## The promotion of traditional values …

In what follows, I will analyse a series of cases where the ROC has acted as moral conservative norm promoter at the international level. The examples support my claim that the church acts as moral entrepreneur and creates ‘traditional values’ in response to the liberal and egalitarian evolution of the international human rights system. In my presentation of the cases, I follow the established categorisation in the literature according to which norm entrepreneurship comprises three levels: (1) norm protagonists, (2) organisational platforms and (3) supportive state actors (Finnemore and Sikkink 1998). The examples are arranged with regard to the level of organisational platform used. This makes sense inasmuch as both the norm promoter and the supporting state remain the same in all of these cases: the ROC and Russia.

### … through the European Court of Human Rights

The ROC has acted as a moral conservative norm promoter through the ECHR in several documented cases (Rimestad 2015). Here I want to look at two: the case *Eweida and others* *v* *the United Kingdom* (ECHR 2013) and the case *Lautsi* *v* *Italy* (ECHR 2011). In the latter case, the Russian state acted as supporting agent.^12^

The *Lautsi* case dealt with the legitimacy of religious symbols in the public sphere. In November 2009 the ECHR ruled that the compulsory display of the crucifix in Italian state schools represented a violation of the European Convention of Human Rights (article 9 on freedom of conscience and religion, and article 2 of the optional protocol on education). The disputed case originated from the complaint filed by a Finnish citizen living in Italy that the display of the crucifix in the classroom attended by the family’s children violated their right to freedom of conscience. The claimant argued that according to the principles of state secularity, explicated through the Italian Constitutional Court in 1989, no religious symbols should be displayed in the public space of a school. In its first verdict in November 2009, the ECHR adopted the point of the view of the claimant, effectively demanding the removal of crucifixes from Italian classrooms. The judgement caused a heated debate both in Italy and internationally on the place of religion in the public space and the power of the Strasbourg Court to interfere with church–state relations in individual countries. Many critics of the verdict felt that the court had, as summarised by the Italian law scholar Marco Ventura, ‘imposed a vision of *laïcité* that is modelled on the pluralistic neutrality of the great Western liberal democracies’ (Ventura 2011), but was not necessarily congruent with the cultural tradition and religious history of the Italian state.

The first *Lautsi* decision evoked a strong reaction by the ROC. Archbishop Ilarion (Alfeev) sent a letter to the Vatican Secretary of State in which he said that the Moscow Patriarchate considered the verdict ‘an attempt to impose radical secularism everywhere despite the national experience of church-state relations’ (ROC 2009a). He added that religious communities in Europe should work together to discuss the fact that ‘the Court has turned into an instrument of promoting an ultra-liberal ideology’ (ROC 2009a). Patriarch Kirill sent a letter to the Italian Prime Minister Silvio Berlusconi in which he stated his ‘full and unconditional support for the intention of the Italian Government to appeal this decision … in cooperation with the Roman Catholic Church’ (ROC 2009b). The ECHR’s judgement convinced the Russian Church’s leadership that ‘on the European continent an encroachment on the religious symbols of Christianity is taking place’ (Russkaya Narodnaya Liniya 2011). Church officials repeatedly mentioned the *Lautsi* case in public interventions between 2009 and 2011 as evidence that ‘aggressive secularism’ and ‘Christianophobia’ were on the rise in Europe (Doerry, Neef, and Schepp 2009; Ilarion 2010; ROC 2010a, 2010b).

The Italian government appealed against the first ruling, supported by the Vatican and a coalition of several countries, namely the Russian Federation, Armenia, Bulgaria, Cyprus, Greece, Lithuania, Malta, Monaco, Romania and San Marino. In March 2011 the ECHR overturned the first ruling and found that it was up to Italy to decide whether there should be crucifixes in Italian public schools (in juridical terms, the court accorded Italy a ‘margin of appreciation’^13^). In its verdict, the ECHR conceded that it was not possible to derive one specific model of ‘admissible’ church–state relations from the European Convention of Human Rights, but instead that ‘every country is free to decide “which place to give to religion” and to favor Christianity, or rather the dominant churches’ (Ventura 2011).

The ROC played an active role in forming a coalition of supporters of the Italian government in the appeal. The coalition of supporters has been analysed by Pasquale Annicchino, who has pointed out that the collaboration of conservative groups from different religions was decisive in turning around the first verdict of the court during the appeal (Annicchino 2011). The particularly important role of Russia and the ROC in the *Lautsi* appeal case was later recognised by the Italian government during a meeting between the Italian Ambassador to Russia and Patriarch Kirill (Interfax Religion 2011).

*Eweida and others v the United Kingdom* was another case brought to the ECHR that was followed closely by the Moscow Patriarchate. This case again involved the wearing of a cross by a British Airways stewardess of Coptic Christian faith, who had decided to wear her baptismal cross around her neck in a visible fashion. She was forced to take unpaid leave from her job in 2006 unless she agreed to wear the cross in a less conspicuous fashion. Even though British Airways eventually identified a regulation under which she could be allowed to wear the cross, the airline refused to pay the wages that the claimant had lost during the period of suspension, and the case was taken to Strasbourg. The representative of the ROC in Strasbourg followed the case closely (REOR 2013a, 2013b, 2013c). He even published a detailed theological statement on the Orthodox view of the wearing of the baptismal cross (Ryabykh and Ponkin 2012).

The two cases show how the ECHR has become an organisational platform for the ROC to promote its vision of Christianity’s privileged position in the European public sphere and create alliances with likeminded state and non-state actors. Furthermore, these examples show that the church can rely on the Russian Federation as a supporting state since it is a member of the Council of Europe.

### … through the United Nations Human Rights Council

The United Nations Human Rights Council (UNHRC) has likewise become another platform where the ROC acts as a norm entrepreneur with the support of the Russian state. One concrete example is the debate on ‘traditional values’, which took place in the UNHRC between 2009 and 2012 (McCrudden 2014). This debate is an excellent example of how the ROC, supported by Russian state diplomacy, promotes its particular traditionalist normative agenda through the organisational platform of the United Nations. This particular case is also interesting because it was, by and large, a failure for the ROC and Russian UN diplomacy. The UNHRC eventually did not issue a document on traditional values and human rights in the spirit of its initial promoters.

On 2 October 2009, the United Nations Commission on Human Rights (UNCHR) was called to decide on a resolution 12/21 ‘Promoting human rights and fundamental freedoms through a better understanding of traditional values of humankind’.^14^ Resolution 12/21 was presented by the representative of the Russian Federation to the Human Rights Council Valery Loshchinin, and it requested ‘to convene, in 2010, a workshop for an exchange of views on how a better understanding of traditional values of humankind underpinning international human rights norms and standards can contribute to the promotion and protection of human rights and fundamental freedoms’ (UNHRC 2009). This resolution was adopted against the votes of the western countries^15^ and, one year later, on 4 October 2010, the requested international ‘workshop’ entitled ‘Traditional Values and Human Rights’ took place at the UNCHR in Geneva. The press service of the Moscow Patriarchate reported extensively on the workshop and the preceding resolution, presenting it as the outcome of Kirill’s address to the General Assembly of the United Nations in March 2008 (Ryabykh 2010b). Among the participants in this workshop was Igumen Filip (Ryabykh), representative of the Moscow Patriarchate in Strasbourg, as already mentioned above in the context of the *Eweida* case. In his speech at the workshop, Igumen Filip (Ryabykh) said that religious views on matters of human rights should be taken into account in the development and establishment of international human rights standards in order to counteract efforts to promote a ‘new generation of human rights’ such as ‘the right to sexual orientation, euthanasia, abortion, experimentation with human nature’ (Ryabykh 2010b).^16^ ‘It is about time that the ideological monopoly in the sphere of human rights is over’, he said, and added: ‘… from the point of view of democracy, it is important to provide an opportunity for representatives from different philosophical and moral views to participate in the development of the institution of human rights’ (Ryabykh 2010b). The representative of the Moscow Patriarchate made a call for the inclusion of religious voices into a post-secular type of deliberation over human rights (I have developed this interpretation at length in Stoeckl 2014, chapter 5).

In March 2011, the Human Rights Council again adopted a resolution, 16/3 of 24 March, entitled ‘Promoting human rights and fundamental freedoms through a better understanding of traditional values of humankind’ (UNHRC 2011). Resolution 16/3 recalls resolution 12/21, welcomes the holding of the workshop just mentioned, decides to remain ‘seized of the matter’ and affirms that ‘dignity, freedom and responsibility are traditional values’. It also notes ‘the important role of family, community, society and educational institutions in upholding and transmitting these values’.^17^ The press service of the Moscow Patriarchate reported that ‘the approval of the resolution continued the Council’s work on traditional values begun on 18 March 2008 by an address of His Holiness Patriarch Kirill of Moscow and All Russia, who was the head of the Department for External Church Relations in the rank of metropolitan, at the UN Human Rights Council’ (ROC 2011). Resolution 16/3 contained the request to the Human Rights Council Advisory Committee to prepare a study on how a better understanding and appreciation of traditional values could contribute to the promotion and protection of human rights, and to present that study to the Council before its twenty-first session.

By the time the twenty-first UNHRC session was convened in September 2012, the study had not been finished, but the rapporteur for the report presented a ‘preliminary study’ (UNHRC 2012a) and tabled a new resolution (21/3), entitled ‘Promoting human rights and fundamental freedoms through a better understanding of traditional values of humankind: best practices’ (UNHRC 2012b).^18^ Resolution 21/3 stated that the Human Rights Council remained seized on the matter of traditional values, it took note of the fact that the Advisory Committee was in the process of preparing the aforementioned study on the topic and it requested an additional study with the aim of ‘collect[ing] information from states members of the United Nations and other relevant stakeholders on best practices in the application of traditional values while promoting and protecting human rights and upholding human dignity’. The Russian press, commenting on resolution 21/3 on ‘best practices’, presented it as a successful joint venture of the Moscow Patriarchate and Russian state diplomacy. The Russian Ministry of Foreign Affairs released a press statement in which it emphasised that the Russian proposal for a resolution on ‘best practices’ in the promotion of human rights through a better understanding of traditional values had passed the Human Rights Council with an ‘absolute majority’ of votes, including votes from countries that belonged to the Organization of Islamic Cooperation and the Arab League. It continued that ‘no state or group of states has the right to monopolize the interpretation of human rights norms’ and concluded that the Russian Federation, together with ‘likeminded partners’, would continue to promote the topic of the ‘inextricable relationship between human rights and traditional moral values’ (MID 2012). This press statement was taken up by the websites of the Moscow Patriarchate and the World Russian People’s Council, which reported the news and added that ‘this position of the Russian Foreign Ministry has been developed in dialogue with the Russian Orthodox Church and other traditional Russian religions’ (ROC 2012; VRNS 2012).

As to the ‘Study of the Human Rights Council Advisory Committee on promoting human rights and fundamental freedoms through a better understanding of traditional values of humankind’, which was finally presented on 6 December 2012, it is noteworthy that, compared to the above-mentioned resolutions, it did not receive the church’s attention or publicity. One reason for this scant attention (and maybe the reason why the Russian diplomats immediately tabled the additional ‘best practices’ study) could be the fact that the study undermines the intention of the original promoters of the resolution which was to promote a traditionalist normative agenda. A comparative reading of the preliminary study prepared by the Russian rapporteur (UNHRC 2012a) and the study which finally came out of the Advisory Committee (UNHRC 2012c) shows great discrepancies between the way in which the topic was promoted by the Russian rapporteur and the consensus inside the Advisory Committee. It is particularly noteworthy that the document by the Russian rapporteur repeats the argument advanced by the ROC that human rights, duties and responsibility to society are linked based on article 29 of the *Universal Declaration of Human Rights*:

“Any society or State, the report states, has a system of ‘law – obligation – responsibility’”, without which the fundamental rights and freedoms of the individual cannot be guaranteed. This close link is underlined in article 29 of the Universal Declaration of Human Rights. (UNHRC 2012a)

But the study signed off by the Advisory Committee basically refutes this statement. Article 29 is not even mentioned, but also the concepts of ‘responsibility’ and ‘duties’ are carefully contextualised in the human rights documents and their applicability is restricted:

[para 30] The African Charter on Human and Peoples’ Rights […] refers to individuals’ duties […] Asian Confucian tradition emphasizes the responsibility of individuals, families and communities in caring for others. Similarly, the preambles to the International Covenant on Civil and Political Rights and the International Covenant on Economic, Social and Cultural Rights recognize that the individual is ‘under a responsibility to strive for the promotion and observance of the rights recognized in the present Covenant’. Thus, although it should be emphasized that human rights are inalienable and inherent in the human person, and are not conditional upon ‘responsible behaviour’, individuals may be regarded as having a responsibility to promote respect for human rights, and not to cause human rights violations against other individuals. […] [para 31] In 2005, the Economic and Social Council voted against a proposal to develop a text on ‘human responsibilities’ owing to concerns that this could undermine the principle of universality. (UNHRC 2012c)

After this study, the ROC and Russian UN diplomacy seem to have abandoned the UNHRC platform for the promotion of traditional values. In the best practices study that was finally presented in 2013 (UNHRC 2013), there is no constructive input from traditionalist actors from Russia. One reason why the initiative faded out in 2013 could be that Russia’s term in the Human Rights Council expired at the end of 2012 (Murphy 2013). The ‘traditional values’ episode highlighted above illustrates how the agenda promoted by the ROC received support from Russian state diplomacy and how the UNHRC was used as a platform for the advancement of the church’s traditionalist norm agenda. Inasmuch as the episode was in the end a failure, it also illustrates that norm diffusion is not always successful. In this particular case, the promoters of the traditional values agenda failed to obtain sufficient support, and what Finnemore and Sikkink described as a ‘norm cascade’ (Finnemore and Sikkink 1998).

### … through the Council of Europe

The Russian Federation became a member of the Council of Europe in 1996. Since then it has signed and ratified a great number of treaties and conventions under the Council of Europe, also the European Social Charter in 2009. Under this charter, which contains basic social rights, Russia is obliged to reform its family law. This reform was developed under President Medvedev and the reform bill was submitted to the State Duma in autumn 2012. At this stage of the legislative process, this seemingly uncontroversial topic turned into a major political issue, which saw the church go up in arms together with other nationalist forces against the ‘imposition of foreign rights standards’ (VRNS 2013b).^19^ Clerics and parents’ associations were particularly upset with the idea that under the new legislation it could become easier for authorities to remove children from parental custody. At a protest rally against this reform, priest Vsevolod Chaplin, responsible for the relations between the Moscow Patriarchate and civil society, declaimed that the reform was imposed by ‘international organisations’. The church, he said, was against the idea that the state should interfere with the educational rights of parents in any way (VRNS 2013b). In February 2013 the Bishops’ Council of the ROC issued a declaration in the same vein, stating that ‘any system of children’s rights should be adapted to national culture and traditions’ (VRNS 2013a). Religious media fanned fears that the reform would lead to children from low-income families being taken away from their parents, thus undermining parental authority and fostering corruption (PRAVOVRNS 2013). A seemingly uncontentious reform had thus transformed into a major controversy and the ROC had identified a new frontier in its fight for traditional values: *yuvenal’naya yustitsiya*.^20^

The ROC’s norm entrepreneurship in this case worked differently from the previous cases. Again both Russian Orthodox and Russian state actors used an international organisation, the Council of Europe, as organisational platform to promote their conservative normative agenda, but this time they acted in response to a domestic, not an international debate. Roughly at the same time as when the Russian public was debating the family law reform, the Russian deputy to the Parliamentary Assembly of the Council of Europe Alexey Pushkov (a member of the United Russia party) tabled a Council of Europe motion entitled ‘The abuse by social services of member States of the Council of Europe of their authority to remove children from their parents’ custody’ (PACE 2012). The motion expressed wariness towards ‘an excessively broad interpretation by social services of their rights’ and demanded that the Council of Europe carry out a thorough analysis of the practice of removing children from parental custody. This motion led to the preparation of a report entitled ‘Social Services in Europe: legislation and practice of the removal of children from their families in Council of Europe member states’ (PACE 2015), published in March 2015. The author was Olga Borzova, a deputy from the Russian Federation and, just like Alexey Pushkov, a member of the United Russia party. Both had in the past voiced publicly their support for the Moscow Patriarchate’s battle against *yuvenal’naya yustitsiya* (PRAVMIR 2011; Smirnov 2013). Borzova’s report to the Council of Europe, which gave an overview of the situation of children that have been placed in state care in Europe and concluded that the removal of a child from parental custody should be a tightly controlled measure applied with utmost scrutiny, was uncontroversial. The recommendations based on Borzova’s report were adopted by the Parliamentary Assembly of the Council of Europe unanimously. The resolution was hailed in Russian conservative media as a sign of the Council of Europe’s support to the fight against *yuvenal’naya yustitsiya*. The Russian newspaper *Kommersant* opened with the headline ‘The Council of Europe stands up against the unjustified removal of children from parents: the Parliamentary Assembly publishes a resolution in defence of family-ties’ (Kommersant 2015) while the patriotic internet portal Russkaya Narodnaya Liniya published the following headline: ‘Council of Europe against *yuvenal’naya yustitsiya*’ (Russkaya Narodnaya Liniya 2015).

I interpret this episode in the Council of Europe as yet another example of the ROC’s norm protagonism. From the very beginning of the debate, the Russian Orthodox Church was very proactive in its opposition to the reform of Russian family law inside Russia. With state support in the form of the two Russian deputies’ active engagement, conservative religious actors managed to use the Council of Europe Parliamentary Assembly as an organisational platform for promoting domestically the message that *yuvenal’naya yustitsiya* was a reform against traditional family values.

### … through nongovernmental organisations

The ROC also acts as moral conservative norm promoter at the level of civil society and through NGOs both inside and outside Russia. In this article, I will look only at the external activities and the involvement of the Russian Orthodox Church in transnational civil society organisations and make a few observations that will be developed in greater detail as part of a future and more extensive analysis.^21^ The examples gathered here indicate a trend, to be examined in future research, that Russian Orthodox norm entrepreneurship also engages non-Russian actors and foreign NGOs, thus creating transnational nongovernmental organisational platforms through which the ROC promotes traditional values.

In the beginning of 2013 public opinion in France was divided on the question of same-sex marriage (*marriage pour tous* in French) and the right of same-sex couples to adopt children. Several hundred thousand people in Paris and other French cities voiced their opposition to the reform bill promoted by President François Hollande giving same-sex couples the same entitlements as heterosexual couples. The protest movement called *La Manif pour tous* united a diverse groups of actors, including the French Roman Catholic Church and the rightist political spectrum. Roughly one year later, on 29 March 2014 the External Relations Department of the ROC reported on the visit of a French *Manif pour tous* delegation.^22^ During the visit, the spokesman of the Patriarchate explained: ‘The members of this delegation believe that Russia is today one of the few countries in the Christian world which […] commits to the protection of the laws of nature and the development of the human person’. The aim of the visit, he said, was ‘to find Russian partners in the fight for traditional values’ (Poujol 2014). The visit, which was reported with some degree of controversy in the French press (La Croix 2014), is only one example of the existing ties between Russian Orthodox traditionalists and western moral conservative actors.

Another example includes contacts between US American Evangelical groups and Russia. On 25 November 2013 the Russian journalist Sergej Sumlennyj published an article about the influence of US American antigay activists on members of the Russian Duma. Reminding the reader of the successful lobbying campaigns against LGBT rights waged by conservative Evangelicals in Eastern Africa, Sumlennyj suggested that Russia was next on the list: conservative antigay rights activists were now testing an ‘African approach to Russia’, and members of the Russian Duma, such as Elena Mizulina, were joining the battle (Sumlennyj 2013). The influence of conservative religious groups from the United States on the legal process that upheld the criminalisation of homosexuality in Uganda has been widely recognised (McCrudden 2015). What is less well known is that one of the American activists, Scott Lively, was involved in promoting the Russian law ‘against homosexual propaganda’. Lively visited Russia several times between 2012 and 2013. On his blog he published an ‘Open Letter to Vladimir Putin’, in which he praised the Russian president for restricting the rights of homosexuals in the public sphere (Lively 2013a). Lively also made contact with the Moscow Patriarchate. In October 2013 he was invited by Archpriest Dmitry Smirnov to speak on his national television programme (Lively 2013b). Smirnov used to be the Patriarchate’s spokesman for military chaplaincy before he moved to a different area, family affairs and engaged, among others, with the Council of Europe and deputy Alexej Pushkov on the issue of *yuvenal’naya yustitsiya* (see above) (Smirnov 2013). The US Evangelical–Russian connection was also the topic of an article published on 7 January 2014 in *The Nation*. The article ‘How US Evangelicals fuelled the rise of Russia’s Pro-Family Rights’ presents some striking evidence on the close connections between US Evangelicals and the ROC, connections that originate in Moscow’s American expat business circles (Federman 2014).

Italian pro-family activists seem well connected with Russia as well. In 2015 the Italian NGO *Pro Vita* (*For Life* in Italian) organised a series of conferences in the Czech Republic, Slovakia and Hungary including the participation of the Russian moral conservative protagonist Aleksej Komov, president of the organisation *Familypolicy.ru* ^23^ and spokesperson of the Commission for the Family of the Moscow Patriarchate. The announcement on the website of *Pro Vita* read as follows:

Central Europe is certainly more sensitive to ethical issues than our spoiled and hedonistic West. 70 years of state-imposed materialism has tried these peoples hard, but they have not put out the light of natural reason – unlike what happened in our decadent societies of rampant consumerism. But those countries now have to suffer the pressure and blackmail of the European Union and the US, in order to adapt the deadly homosexual agenda, which the West has followed for decades. (Pro Vita 2015)

These three examples drawn from the sphere of traditionalist transnational civil society – the visit to Russia by the French *Manif pour tous* delegation, the Russia connections of the American activist Scott Lively and the cooperation of the Italian NGO *Pro Vita* and *Familypolicy.ru* – can be interpreted in the theoretical framework of international norm entrepreneurship. The organisational platforms used by Russian Orthodox actors are, in these cases, NGOs and their instruments of communication: blogs, news reports, manifestos, and joint conferences and workshops. In the cases gathered here there is no direct involvement by the Russian state as facilitator or supporter since the activities are independent from the state and confined to NGOs. Nevertheless, the above-mentioned cases are examples of the norm protagonism of Russian Orthodox actors in the international arena.

## Conclusion

What are the traditional values promoted by the Moscow Patriarchate? From the cases presented in this article and from the literature (Stepanova 2015; Agadjanian forthcoming) it is possible to derive the content of ‘traditional values’: visibility of Christian symbols in the public sphere, opposition to all forms of lesbian–gay–bisexual–transgender rights, restrictions on the breadth of women’s and children’s rights, and opposition to abortion, euthanasia, reproductive and stem cell research, as well as the defence of believers’ rights of religious expression, i.e. battle against any free speech that can amount to blasphemy. Although the term ‘traditional values’ suggests continuity with the past and rootedness in established practice, the theoretical toolkit of social constructivism in IR, the short history of the Russian Orthodox ‘discovery of article 29’ and the cases of rights advocacy explored in this article open up a different interpretation: ‘traditional values’ are a label given to practices and ideas that have hitherto not been named or ‘dramatised’ as normatively relevant, but have become normatively relevant because of the liberal and egalitarian evolution of the international human rights system and its impact on domestic politics. Therefore, the traditional values agenda is the conservative flipside of the progressive human rights system.

Beyond the constructivist perspective and beyond the rights advocacy agenda, traditional values also have a substantive content, which Christopher McCrudden has summarised as: (1) values of national sovereignty; (2) religious practice; (3) the ‘traditional societies’ of the global south and (4) philosophical conservatism with its emphasis on particularism, context and complexity (McCrudden 2014). The ROC has made itself the spokesperson of a traditional values agenda: (1) asserting Russian national legal sovereignty; (2) defending Orthodox Christianity; (3) forming coalitions with traditionalist forces from the rest of the world, including the global south; and (4) promoting philosophical conservatism, including the Russian religious philosophical tradition.

However, it is noteworthy that the norm protagonism of the Moscow Patriarchate in the international arena draws on these substantive sources selectively and via issue coalitions that bracket existing conflicts. For example, the ROC joined forces with American Evangelicals in opposition to gender rights, but it did not support their approach to religious freedom inside Russia. The ROC collaborated with the Roman Catholic Church on family values, but this does not automatically entail progress in doctrinal ecumenical dialogue. Coalitions with countries from the global south, for example, at the level of the United Nations, have not meant that the ROC has endorsed their battle for global economic equality. It is a task for future research to find out in more detail how this selective agenda setting and coalition making takes place and what it can tell us about ideological factions and power balances inside the ROC.

Inasmuch as the traditional values agenda understands itself as a corrective measure to the progressive human rights system, one should acknowledge its capacity to raise pressing questions on the definitions of human rights that go beyond a sharp political confrontation with specific issues, such as LGBT rights and abortion. The larger issues that stand behind these embattled rights are debates over the definition of life, bodily integrity, personal freedom and the power of the state, as well as debates on the quality of democracy, collective rule-making and democratic accountability of international institutions. It is beyond the scope of this article to go into depth on these issues, but it is important to recognise that the norm protagonism of the ROC is one element in a more general debate on the features of what Jürgen Habermas has called ‘postsecular society’.^24^

The aim of this article was to explore the ideological prerequisites and the practical channels of the moral norm protagonism of the ROC. The theoretical focus on moral norm entrepreneurship has provided a useful lens for understanding the role of the ROC in international human rights politics. The findings from this article demonstrate a degree of independent agency of the ROC, which is frequently overlooked by studies that subsume the traditional values agenda under Russia’s value-based foreign policy (Curanović and Leustean 2015; Anderson 2015; Laruelle 2015). It thereby adds one facet to the growing field of research that analyses Russian actors beyond the East–West confrontation and in the context of a global norm contestation.

## Notes

1. This article marks the beginning of a research project on the role of the Russian Orthodox Church in transnational morality politics. The project *Postsecular Conflicts* (ERC-2015-StG-676804, principal investigator: Kristina Stoeckl) will undertake a comprehensive investigation and interpretation of the ‘traditional values’ agenda and networks. To follow this research, please go to https://www.uibk.ac.at/projects/postsecular-conflicts/.

2. The term ‘norm entrepreneur’, as it is used in the body of IR theory I refer to, should be distinguished from the concept of ‘entrepreneur’ or ‘social entrepreneurship’ in social network literature, where the entrepreneur is defined as someone ‘who tries to manipulate norms and relationships for his own social and psychological benefit’ (Boissevain 1974, 7). The issue of motivation (‘benefit’) and of the nature of interaction (‘manipulation’) raises a different set of questions, such as ‘why does the ROC promote certain norms and not others?’, ‘what are the benefits and costs of norm protagonism for the Church?’, ‘is the interaction of the ROC with other actors “sincere” or “manipulative”?’ I will not deal with these questions here because the data on which I have based my argument in this article do not provide the necessary information to answer them here. For further details on the ongoing research, see note 1.

3. In this article, I use ‘norm entrepreneur/norm entrepreneurship’ and ‘norm protagonist/norm protagonism’ interchangeably. In the Russian linguistic context, which is relevant for this research, the connotations of ‘entrepreneur’ (*businessmen, oligarchs*) are an obstacle for a correct understanding of the argument and translation. In this context, the term ‘norm protagonism’ is preferable. (I would like to thank the anonymous reviewer for having me made aware of this point.)

4. The threefold division of the ROC’s internal ideological spectrum is an analytical and heuristic tool which does not correspond to actors’ self-descriptions. There can be overlaps between the categories: for example, traditionalists and fundamentalists tend to converge on moral and ethical positions, but they differ on the political strategies they derive from this conviction.

5. There is one exception to this statement and that is the role of militant Russian Orthodox activists and Russian nationalists in the conflict in eastern Ukraine. There we find that Orthodox fundamentalism has indeed become politically cogent as part of an international conflict, despite a more cautious stance by the church leadership.

6. The Russian domestic political situation is another important context for understanding the traditional values agenda of the ROC in the international arena. While I do not discuss the domestic situation here for reasons of space, it is important to recognise that traditional values have become increasingly politicised inside Russia in terms of antiliberalism, antiwesternism and Russian exceptionalism. See Agadjanian (forthcoming) and Stoeckl (2014, 92–102).

7. In this article, I use ‘ROC’, ‘Moscow Patriarchate’ and ‘church leadership’ interchangeably. This is a terminological choice in the context of my argument where I focus on external church relations and the official ideological position of the present Moscow Patriarchate as expressed in its external affairs. This terminological choice does not in any way dispute the pluralism of ideological positions inside the ROC.

8. (1) Everyone has duties to the community in which alone the free and full development of his personality is possible. (2) In the exercise of his rights and freedoms, everyone shall be subject only to such limitations as are determined by law solely for the purpose of securing due recognition and respect for the rights and freedoms of others and of meeting the just requirements of morality, public order and the general welfare in a democratic society. (UDHR 1948, emphasis added)

9. The traditional values argument, based on article 29, is an alternative to the (inconclusive) traditional family values argument in terms of ‘collective rights’ (Stoeckl 2014, 83–84). However, both strategies of advancing traditional values to set limits to individual human rights should be interpreted as part of a general pattern, prevalent in Russian social history, according to which the individual is sharply opposed and generally subordinated to the collective (see Kharkhordin 1999).

10. In his speech in front of UNESCO in 2007, Kirill appealed to ‘those people in the world who share traditional moral values founded on religion’ (Interfax Religion 2007), suggesting that traditional values have their roots in all world religions.

11. The same argument is made by the ROC with regard to the ‘limitation clauses’ of the European Convention of Human Rights.

12. This section follows closely a prior analysis in Stoeckl (2014, 114–117).

13. The term ‘margin of appreciation’ refers to the discretionary space that national authorities hold vis-à-vis the ECHR in fulfilling their obligations under the European Convention on Human Rights. It is a mechanism that allows for the recognition of diverse cultural and legal traditions embraced by each member state of the Council of Europe.

14. In this section, the analysis of the resolutions from 2009 to 2011 follows a prior analysis in Stoeckl (2014, 109–113). The interpretation of developments from 2012 onwards is based on new material.

15. Voting results of resolution 12/21: In favour: Angola, Bahrain, Bangladesh, Bolivia, Burkina Faso, Cameroon, China, Cuba, Djibouti, Egypt, Gabon, India, Indonesia, Jordan, Kyrgyzstan, Madagascar, Nicaragua, Nigeria, Pakistan, the Philippines, Qatar, Russian Federation, Saudi Arabia, Senegal, South Africa and Zambia. Against: Belgium, Chile, France, Hungary, Italy, Japan, Mauritius, Mexico, the Netherlands, Norway, Republic of Korea, Slovakia, Slovenia, the UK and the US. Abstaining: Argentina, Bosnia and Herzegovina, Brazil, Ghana, Ukraine and Uruguay.

16. Igumen Filip (Ryabykh) seems to have misrepresented the expansive nature of the human rights system here. There is no ‘right to sexual orientation’, but the right to nondiscrimination on grounds of sexual orientation.

17. Voting results of resolution 16/3: In favour: Angola, Bahrain, Bangladesh, Burkina Faso, Cameroon, China, Cuba, Djibouti, Ecuador, Ghana, Jordan, Kyrgyzstan, Malaysia, Maldives, Mauritania, Nigeria, Pakistan, Qatar, Russian Federation, Saudi Arabia, Senegal, Thailand, Uganda and Zambia. Against: Belgium, France, Hungary, Japan, Mauritius, Mexico, Norway, Poland, Republic of Korea, Slovakia, Spain, Switzerland, the UK and the US. Abstaining: Argentina, Brazil, Chile, Guatemala, Republic of Moldova, Ukraine and Uruguay.

18. Voting results for resolution 21/3: In favour: Angola, Bangladesh, Burkina Faso, Cameroon, China, Congo, Cuba, Djibouti, Ecuador, India, Indonesia, Jordan, Kuwait, Kyrgyzstan, Libya, Malaysia, Maldives, Mauritania, the Philippines, Qatar, Russian Federation, Saudi Arabia, Senegal, Thailand and Uganda. Against: Austria, Belgium, Botswana, Costa Rica, Czech Republic, Hungary, Italy, Mauritius, Mexico, Norway, Poland, Romania, Spain, Switzerland and the US. Abstaining: Benin, Chile, Guatemala, Nigeria, Peru, Republic of Moldova and Uruguay.

19. In this section, the analysis of the debate until mid-2013 follows my book (Stoeckl 2014, 98–99), while the interpretation of developments from mid-2013 onwards is based on new material.

20. The term not only sounds foreign in Russian, it is also a completely misleading translation of the English term ‘juvenile justice’, which means criminal law vis-à-vis minors and not, as it was used in the Russian debate, family law.

21. This will be part of the research project *Postsecular Conflicts*, see note 1.

22. The visiting members of the French delegation who were received in Moscow by the head of the External Relations Department Metropolitan Ilarion included the French Bishop of Bayonne and Oloron Marc Aillet; the director of the European Centre for Law and Justice Grégor Puppinck; the secretary general of the French ‘Alliance Vita’ and one of the organisers of the *Manif pour tous* Caroline Roux; the journalist of the Catholic weekly *Famille Chrétienne* Aymeric Pourbaix; and the secretary general of the pro-life Foundation Jérôme-Lejeune Thierry de la Villéjegu (ROC 2014).

23. Familypolicy.ru was the official host organisation of the Seventh World Congress on Families in September 2014 organised in cooperation with the Howard Center for Family, Religion and Society from Rockford, Illinois. The American partner organisation pulled out after sanctions were put in place against Russia in connection with the annexation of Crimea. The organisation of this congress is another example of Russian Orthodox norm entrepreneurship that requires further detailed analysis, which goes beyond the scope of this article.

24. See note 1 and an article on why moral conservative norm protagonism is a challenge for political liberalism (Stoeckl 2016).
